# A decision support system to follow up and diagnose primary headache patients using semantically enriched data

**DOI:** 10.1186/s12911-018-0679-6

**Published:** 2018-11-13

**Authors:** Gilles Vandewiele, Femke De Backere, Kiani Lannoye, Maarten Vanden Berghe, Olivier Janssens, Sofie Van Hoecke, Vincent Keereman, Koen Paemeleire, Femke Ongenae, Filip De Turck

**Affiliations:** 1 IDLab, Ghent University - imec, Technologiepark 15, Ghent, 9052 Belgium; 2 Department of Neurology, Ghent University Hospital, Corneel Heymanslaan 10, Ghent, 9000 Belgium

**Keywords:** Primary headache disorders, Decision support system, White-box predictive modeling, Mobile cross-platform development, Web application development, Prior knowledge incorporation

## Abstract

**Background:**

Headache disorders are an important health burden, having a large health-economic impact worldwide. Current treatment & follow-up processes are often archaic, creating opportunities for computer-aided and decision support systems to increase their efficiency. Existing systems are mostly completely data-driven, and the underlying models are a black-box, deteriorating interpretability and transparency, which are key factors in order to be deployed in a clinical setting.

**Methods:**

In this paper, a decision support system is proposed, composed of three components: (i) a cross-platform mobile application to capture the required data from patients to formulate a diagnosis, (ii) an automated diagnosis support module that generates an interpretable decision tree, based on data semantically annotated with expert knowledge, in order to support physicians in formulating the correct diagnosis and (iii) a web application such that the physician can efficiently interpret captured data and learned insights by means of visualizations.

**Results:**

We show that decision tree induction techniques achieve competitive accuracy rates, compared to other black- and white-box techniques, on a publicly available dataset, referred to as migbase. Migbase contains aggregated information of headache attacks from 849 patients. Each sample is labeled with one of three possible primary headache disorders. We demonstrate that we are able to reduce the classification error, statistically significant (*ρ*≤0.05), with more than 10% by balancing the dataset using prior expert knowledge. Furthermore, we achieve high accuracy rates by using features extracted using the Weisfeiler-Lehman kernel, which is completely unsupervised. This makes it an ideal approach to solve a potential cold start problem.

**Conclusion:**

Decision trees are the perfect candidate for the automated diagnosis support module. They achieve predictive performances competitive to other techniques on the migbase dataset and are, foremost, completely interpretable. Moreover, the incorporation of prior knowledge increases both predictive performance as well as transparency of the resulting predictive model on the studied dataset.

## Background

### Introduction

Headache disorders are an increasingly recognized health issue in modern society, causing a substantial burden both at personal and societal level [1, 2]. The fact that headache disorders have been underestimated and undertreated globally has been acknowledged by the World Health Organization [3]. In Europe, more than 50% of the people suffer from a headache attack at least once a year [4] and they are the third most expensive neurological condition, after dementia and stroke [5]. Three main classes of headache disorders are recognized. The first class are the primary headache disorders, in which no underlying pathology can be identified, such as trauma or infection. The main subdivisions of the primary headaches disorders are migraine, tension-type headache and trigeminal autonomic cephalalgias (TAC). Of the TACs, cluster headache is the most prevalent type [6]. Secondly, all headaches with an underlying pathology or that can be defined as a symptom of an underlying disease, are called secondary headaches. Cranial neuralgias and facial pain form a third class of headaches. Primary headaches, especially migraine, account for the vast majority of headache burden [7]. According to the 2016 Global Burden of Disease Study migraine is the second leading cause of Years Lived with Disability, and ranks 16th on Disability Adjusted Life Years, which measures health loss due to both fatal and non-fatal disease burden [8, 9].

Proper management of a primary headache disorder requires a correct diagnosis. Often, patients keep track of their attacks in some form of headache journal. A plethora of variables such as the intensity and duration of the attack, associated symptoms and whether or not any triggers are applicable, must all be recorded for a certain time span. The gold standard for headache classification is the International Classification of Headache Disorders (ICHD) [10]. This is a large document containing the different diagnostic criteria for each of the separate headache disorders. Despite advances in recent years, many patients still face diagnostic delay as has been shown for both migraine [11–13] and cluster headache [14–16]. It is only based on a specific and correct diagnosis that appropriate management can be initiated which may include trigger management as well as acute and preventive drug treatment. The patients have to keep track of their headache attacks/days and use of medication on a paper calendar, which physicians then use to adjust the drug treatment accordingly.

Due to aforementioned reasons, a considerable amount of a neurologist’s time is spent on diagnosing and following up headache patients, while especially the latter task could be performed by first-line health-care providers in order to reduce health-care costs [17]. Moreover, due to the nature of the diagnosis process for the more common types of headache disorders, i.e. finding the best match between criteria listed in the ICHD document and data collected from the patient, a machine learning technique could offer a significant added value. To increase the efficiency of both the diagnostic and follow-up phase in the treatment process of primary headaches, a decision support system, composed of three components and a shared back-end, is presented in this paper. A first component is a mobile application that replaces the diary that patients use to keep track of their headache attacks and medicine consumption. The use of this application is two-fold: it enables the collection of the required variables to formulate a diagnosis and it allows the user to record his headache attacks or medicine consumptions during the follow-up phase. In the smartphone era, such a mobile application is more efficient and user-friendly than the use of a paper calendar [18], since the user can now register information concerning his/her attacks at any time and at any place. Furthermore, it could alleviate the need to schedule a first appointment where the calendar system is explained by the physician to the patient. We present a web application as a second component, enabling physicians to consult data corresponding to a specific patient in the form of different visualizations, allowing for efficient interpretation. The third and final component provides clinical decision support by applying supervised machine learning techniques on data collected by the mobile application, which is semantically annotated in the back-end to increase interpretability and predictive performance. Moreover, expert knowledge, defined in knowledge bases is incorporated into different steps of the machine learning algorithm to increase transparency. The resulting model can both guide the physician or mobile application with the queries they pose to patients and serve as clinical decision support during the diagnostic phase.

The contributions of this paper are two-fold. First and foremost, we present a proof-of-concept of an end-to-end system which, to our belief, could drastically increase the efficiency of current treatment processes for primary headache disorder patients. Second, we demonstrate the added value of semantically enriching data, especially in critical domains such as health-care, by presenting two experiments with positive results. The remainder of this paper is structured as follows. In the following subsection, related work is discussed concerning other mobile headache diaries and applications for decision support in the primary headache disorder domain. In “Methods” section, the general platform architecture is presented and each of the components is discussed. Moreover, we present the setup for three experiments, discussed in “Results” section. In a first experiment, we compare different supervised classification techniques on a publicly available dataset containing information about headache disorders and their corresponding classification. Second, we evaluate several over-sampling techniques, that generate artificial samples such that the class distribution becomes more uniform in order to combat the data imbalance problem, which occurs when a classifier favors the majority class. Finally, we investigate different data- and knowledge-driven feature extraction algorithms, based on similarity metrics to each of the class concepts. The goal of these feature extraction techniques is to create extra variables that could help the machine learning classifier in achieving higher predictive performances. We discuss our results, and their implications, in “Discussion” section and conclude our paper in “Conclusion” section.

### Related work

It can be hard to get a clear and high-quality clinical picture of a patient from a consultation alone. Therefore, some form of self-monitoring is preferred, where the patient keeps track of his or her headache attacks over time [19–21]. Clearly, a mobile application is more user-friendly than a paper calendar [22], since it allows patients to register information at any time or place, without having to worry about losing the calendar or forgetting to bring it to a consultation. Quite some mobile headache diary journal applications are already commercially available [23]. The most popular ones, in terms of number of downloads and rating in the Android Play and Apple App Store, include Migraine Buddy [24] and Headache Diary Lite/Pro [25]. Unfortunately, while many solutions exists for patients to keep track of all headache information, the number of solutions that allow physicians to efficiently interpret all collected data is very limited. Most mobile applications provide an export functionality, which allows users to print out a certain representation of their data, which can be brought to a consultation. This is still archaic, and does not solve the problem that patients can forget to bring this printed version to a consultation. Moreover, a physician can only analyze the data, of which the representation is completely determined by the mobile application developers, when the data is provided to him by the patient. A custom-made application that visualizes all collected data allows the physicians to analyze patient data anytime they want, and allows them to tailor the data representation to their own needs [26, 27].

A few researchers have already shown the potential machine learning techniques can offer in diagnosing a headache disorder. In Keight et al. [28], nine different classifiers were compared on a dataset consisting of 836 primary headache cases, each containing 65 different variables. Each case is labeled as one of five classes (tension-type, chronic tension-type, migraine with or without aura and trigeminal autonomic cephalalgia), collected from two Turkish medical institutions. They show that a stacking classifier achieves the best predictive performance, at a cost of having very limited interpretability. The power of ensembles for headache classification has also been confirmed by Jackowski et al. [29]. Krawczyk et al. [30] present a taxonomy of headache disorders, along with corresponding diagnosis criteria from the ICHD document. They compare 6 different classifiers and three feature selection techniques with each other, and with the performance of a physician, on a labeled dataset of 579 subjects consisting of three classes (migraine, tension-type and cluster headache). They show that reducing the feature set can increase the predictive performance, and that the automated feature selection techniques selects a better subset of features than a physician in terms of resulting predictive performance. Moreover, they show that the predictive performance of C4.5, a decision tree induction algorithm, closely matches the performance of black-box counterparts. Celik et al. [31] introduce an artificial immune algorithm that achieves high predictive performance on a dataset of 849 samples with three classes (migraine, tension-type and cluster headache). The dataset is made publicly available and is used in this study to allow for comparison with their and possible future studies. Furthermore, they present a web-based application that allows for patients to register information concerning their headache attacks and for physicians to consult this data. In 2017, an extension was released, in which they evaluated an ant colony optimization algorithm on their dataset. More importantly, they give a clear overview of all prior research for primary headache disorder classification [32]. Yin et al. [33] propose a rule-based and case-based reasoner, which is an extension on a former proposed system [34], and show that these reasoners outperform machine learning classifiers in terms of both precision and recall on their dataset. Finally, it is shown by Garcia-Chimeno et al. that ensemble techniques combined with feature selection can drastically improve predictive performances for headache classification [35], confirming the findings of Jackowski et al. and Keight et al. While the discussed papers provide interesting insights of different methodologies applied to headache disorder classification, none of these, except for research by Celik et al., uses a publicly available dataset or discusses and end-to-end application with components for both patient and physician.

As opposed to Celik et al., we advocate the use of a white-box approach since interpretability and transparency are important factors to boost the physicians trust in the decision support system. To stimulate transparency, we incorporate existing expert knowledge of the headache diagnosis disorder domain into the different phases of our machine learning approach. This is in contrast with a purely data-driven method, where existing knowledge is completely neglected. This hybrid mix of both knowledge-driven and data-driven techniques has other advantages than better interpretability alone. It requires a lot less labeled data and is often faster than the expensive training phase from data-driven methods. On the downside, the predictive performance of the resulting model depends entirely on the quality of the incorporated knowledge [36–38]. Fortunately, expert knowledge in the headache disorder domain is of high quality and can easily be encoded in a machine-interpretable format, as has been shown by Yin et al. The added value of prior knowledge incorporation in the different steps of a machine learning pipeline, for medical tasks in different domains, has already been demonstrated by multiple other studies [39–41].

## Methods

### General overview

**Fig. 1:**
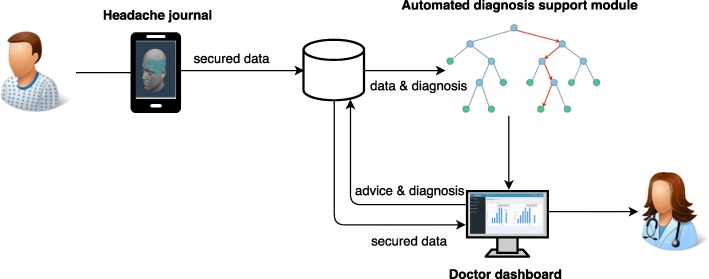
An overview of the different components of the proposed decision support system and how they interact. Icons are taken from www.draw.io & www.iconarchive.com and licensed for non-commercial use

A general overview of the proposed decision support system can be found in Fig. 1. As can be seen, the system is composed out of three main components, with a shared back-end. First, a cross-platform mobile application that allows the patients to register all information concerning their headache attacks in a user-friendly manner. Second, a web application which enables physicians to efficiently process all data collected by the mobile application. Third, we present an automated diagnosis support module to induce an interpretable predictive model from the collected data in order to support the physician in making the correct diagnosis.

### Cross-platform mobile headache journal

**Fig. 2:**
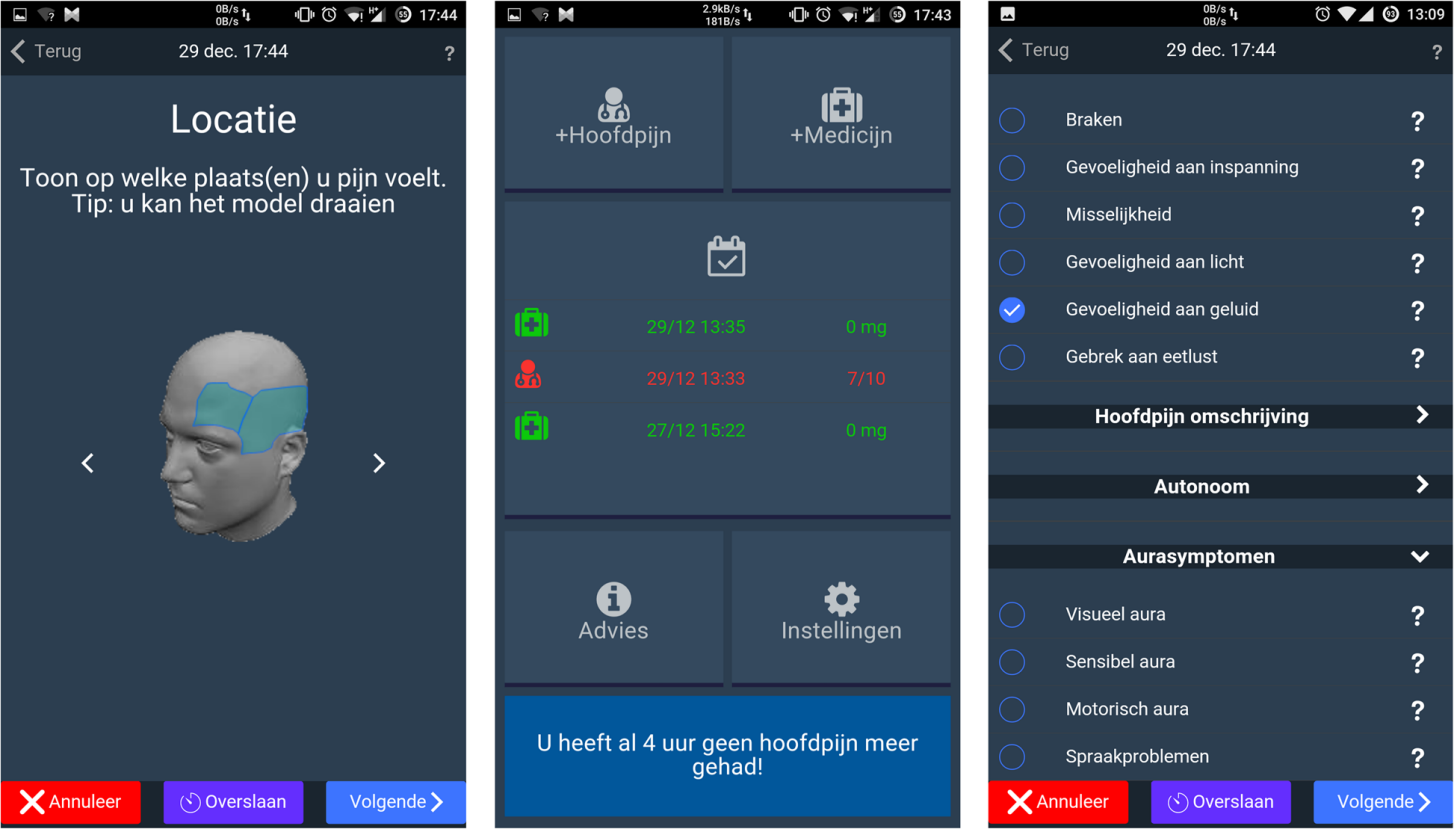
Screenshots of the developed mobile headache journal, Chronicals. On the left screen, the user can select the appropriate location of the headache (translation: “Location. Indicate on which places you feel pain. Hint: you can turn the model.”). The middle screen depicts the home screen and contains a button to add new headache information, add new medicine information, get an overview of registered information, display advice from physicians and configure the settings of the application. On the right screen, the user can select the relevant symptoms for a headache (translation list entries from top to bottom: “vomiting, sensitive to exertion, nausea, sensitive to light, sensitive to sound, lack of appetite, visual aura, sensible aura, motoric aura, speech problems”)

In order for a mobile application to replace the current paper calendars, it had to fulfill a list of requirements, which was composed in consultation with neurologists (authors VK and KP). First, a large number of the patients in a hospital should be able to use the application. Therefore, the application has to run on at least Android and iOS in order to cover the largest fraction (99.8% worldwide [42]) of the market share. Furthermore, in order to minimize development time and future maintenance time, a cross-platform solution should be preferred. Second, it needs to collect all relevant data for the diagnostic phase and this data has to be exportable in an open format such that other software tools can access and use this data. Third, the interface has to be intuitive and take into account the fact that users are often suffering from a headache at the time they will be interacting with the application. As an example, the graphical interface should not be too bright, since a lot of headache patients have photophobia. However, none of the available applications fulfill all the listed requirements. Hence, we developed our own application, called Chronicals, using PhoneGap [43], which allows for the application to be deployed on Android, iOS and Windows Phone. Screenshots of the application can be found in Fig. 2. Since the application has been evaluated in the University Hospital of Ghent (Belgium), all text is in Dutch. The data collected by the application is stored locally on the phone in an encrypted manner and securely sent to the server for subsequent analysis by the physicians, once new data and an Internet connection are available.

### Diagnosis support module

**Fig. 3:**
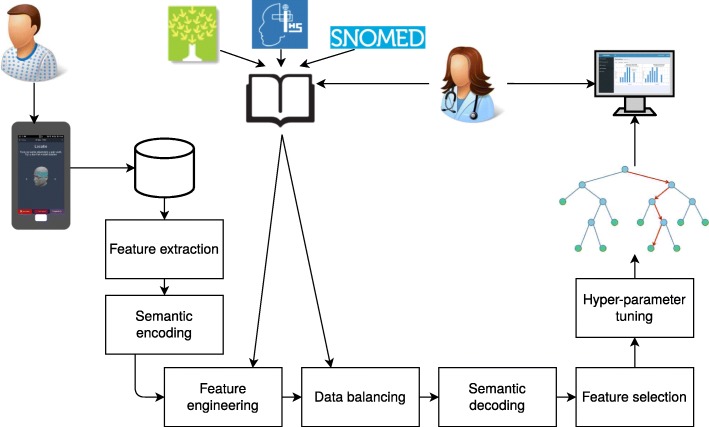
An overview of the automated diagnosis support module. Icons are taken from www.draw.io & www. iconarchive.com and licensed for non-commercial use

One of the most important modules of the proposed decision support system is an automated diagnosis support module. In this module, an interpretable predictive model is generated from the data collected by our mobile application, using supervised classification. Supervised classification is a sub-domain of machine learning in which we try to find a hypothesis, or model, which maps an input vector to one of *K* discrete classes, by the use of labeled examples [44]. The entire flow of the automated diagnosis support module is depicted in Fig. 3. The data collected from patients, by means of our mobile application, is stored in a back-end, which is shared with the web application for the physicians. Additionally, a knowledge base is constructed using expert knowledge, the ICHD document and ontologies such as SNOMED [45]. Both the collected data and the prior knowledge is used to generate feature vectors which are fed to the machine learning technique. Before feeding them, the class distribution in the training dataset is balanced in order to make it more uniform; feature selection is applied using a genetic algorithm [46], which decreases the model complexity and consequently the generalization capability; and the different hyper-parameters of the decision tree induction algorithm, such as the split criterion and the maximum tree depth, are tuned. Each component in the pipeline is now discussed more in depth subsequently.

#### Feature extraction, semantic encoding and decoding

In order to generate feature vectors for the machine learning classification technique, the system first groups and aggregates all the data per patient to form feature vectors. Examples of values in these vectors could be the average or maximum intensity of the attacks, the most occurring locations, a probability of occurring symptoms and so on. Next, the feature vectors are encoded in the form of knowledge graphs [47]. Knowledge graphs are data structures that efficiently and intuitively encode different entities and relationships between them. They can be represented by a set of (subject, predicate, object)-triples, where each triple corresponds to two nodes and a connecting edge in the knowledge graph. The most well-known standardized syntax for representing these triples is called Resource Description Framework (RDF). To transform these numerical feature vectors to knowledge graphs, each (property, value)-pair from the original feature vector is translated to a corresponding triple. An example of an annotated sample of our dataset can be found in Listing 1. All medical concepts, such as the indicated symptoms occurring during the headache attack, are linked with SNOMED (using the owl:sameAs property), which is updated frequently, allowing our knowledge base to be updated if new discoveries are made within the headache disorder domain. These constructed knowledge graphs are used in the data balancing and feature engineering steps of the machine learning pipeline in our proposed system, which are discussed subsequently. Since currently existing machine learning techniques cannot deal directly with semantic data, a method to convert a knowledge graph back to a numerical feature vector is required as well.


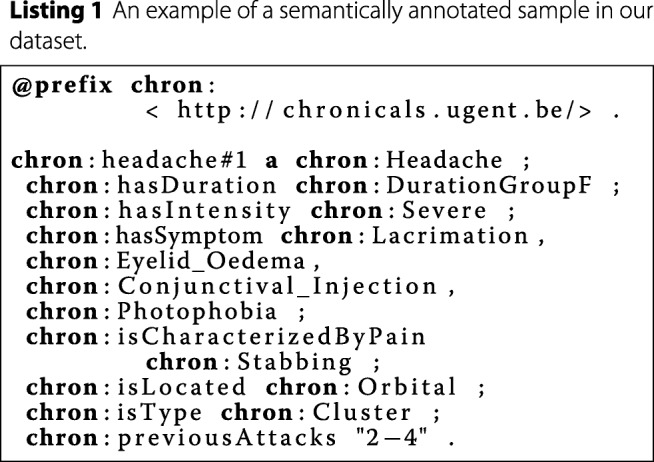


#### Feature engineering

After translating our numerical vectors to knowledge graphs, we augment our dataset by engineering extra features. One possibility are similarity scores to each class concept, based on the principle of a K-Nearest Neighbors (KNN) classifier. The use of such similarity measures is two-fold. One the one hand, they are generic features that can be added to each classification dataset in order to possibly enhance the predictive performance. On the other hand, when the number of deviations between the characteristics of the headache attacks of a certain patient and the diagnostic criteria of the ICHD document is rather high, an alert can be generated to indicate that the physician should pay special attention to the diagnosis of that specific patient.

We can define two categories of techniques to calculate these similarities. First, we can compute these similarities between the feature vectors and a class concept vector, using metrics such as Radial Basis Function (RBF) or cosine similarity. To construct these class vectors, we can calculate the medoid or centroid of all samples belonging to that specific class. Second, we can calculate similarities between the knowledge defined for each class in our knowledge base and each annotated sample within our dataset. Since both the knowledge and sample are represented in the form of a graph, we cannot apply the same metrics as in the aforementioned data-driven approach. One way to define similarity between graphs is through applying graph kernels [48]. Unfortunately, these techniques cannot be applied directly to knowledge graphs encoded in RDF, since these are directed graphs which possess named edges. Lösch et al. introduced graph kernels specifically for RDF data [49]. Moreover, a fast approximation of the Weisfeiler-Lehman (WF) kernel, which achieves state-of-the-art results, has been proposed by de Vries et al. [50]. The WF kernel efficiently counts the equivalent subtrees of depth *d*, by means of an iterative relabeling algorithm. This knowledge-driven approach can be applied in an unsupervised manner, which is a significant advantage.

#### Data balancing

The headache disorder domain is very imbalanced. Migraine headaches are far more common than, for example, cluster headaches. This imbalance is reflected in the migbase dataset as well, which is used in the experiments discussed further, since the fraction of samples labeled with migraine, tension or cluster is 71.73*%*, 21.67*%* or 6.60*%* respectively. This imbalance in the dataset can significantly compromise the predictive performance of the resulting classifier [51]. One way to combat this problem is by generating artificial samples, using the feature distributions of the minority classes reflected in the data, which is called over-sampling. Two prominent over-sampling techniques are Synthetic Minority Over-sampling TEchnique (SMOTE) [52] and ADAptive SYNthetic sampling approach for imbalanced learning (ADASYN) [53], but these are entirely data-driven. In critical domains, such as health-care, a lot of prior expert knowledge about the target domain is often available which is completely neglected by these data-driven techniques. Therefore, we semantically encoded the ICHD document into Web Ontology Language (OWL)-files and generated artificial data that complies to this predefined knowledge. An example of a fragment of such an OWL-file can be seen in Listing 2. Generating these samples is straight-forward: depending on the type of restriction, we sample from the possible choices (e.g. pick one if the restriction is owl:oneValueFrom). The advantage of this knowledge-driven approach is that, as opposed to SMOTE or ADASYN, no data is required since it only depends on the knowledge base. Furthermore, the method is fast, since it just needs to sample values according to the given knowledge for each of the features. An example of an artificial sample, in RDF format, can be seen in Listing 1. These samples can be transformed easily into feature vectors, since the property and object of each triple represent the feature and value respectively.

#### Machine learning classification technique

The existing supervised machine learning models can be divided into two large categories. On the one hand, there are black-box models, such as (artificial) neural networks and support vector machines. These often achieve excellent predictive performances but at a cost of having minimal to no interpretability. While techniques exist that are able to generate dependency plots between small subsets of variables or an instance-based explanation about why a certain prediction was made by the black-box model, such as LIME [54], SHAP [55] and MFI [56], it cannot give a global model-based explanation. On the other hand, there are white-box models which achieve predictive performances that tend to be lower than their black-box counterparts, but posses excellent interpretability since they are able to give both instance- and model-based explanations. Moreover, the instance-based explanations are often more comprehensible and accurate than those generated by the previously enumerated ‘explanation’ techniques. In order to lower the threshold of acceptance within critical domains, the experts should have insights into how conclusions are reached by the underlying model. Therefore, a white-box model seems ideally suited. Examples of white-box techniques include Bayesian networks, ordered rule lists and decision trees. Bayesian networks can be very computationally intensive, making their use impractical in big data settings. An ordered rule list seems like an ideal fit since they are very analogue to the ICHD document, which forms the basis of diagnosing headache disorders. Decision trees are closely related to ordered rule list, as every path from the root to a leaf in the decision tree corresponds to a rule. The advantage of decision trees over ordered rule lists is that it is easier to grasp the model globally, because of the tree structure as opposed to a sequential structure. Moreover, the tree structure provides another advantage: the decision process of the physician or the questionnaire in the mobile application can be optimized such that the maximum number of questions posed to, or tests performed on the patient is equal to the depth of the tree. Therefore, a decision tree induction algorithm was chosen to construct the predictive model for the automated diagnosis support module.

#### Feature selection and hyper-parameter tuning

Incorporating uninformative features in the predictive model both increases the model complexity and can confuse the classifier, leading to a detriment in predictive performance. Therefore, prior to fitting a model on the training data, these uninformative features should be discarded. One way to do this, is by applying a genetic algorithm in which each individual’s genotype is presented by a binary vector, corresponding to a subset of the total feature set. The genetic algorithm efficiently tries to fit instances of the classifier on different combinations of features (individuals), measuring the predictive performance after each fit. After each iteration (or generation), different individuals are merged together (cross-over) based on their predictive performance (fitness). Moreover, each individual can be mutated with a certain probability in each generation, by flipping bits in the binary vector.

Next to the used feature set, the hyper-parameter setting of the machine learning technique has an impact on the resulting predictive performance of the model as well. For most decision tree induction algorithms, the number of hyper-parameters (such as maximum depth and split criterion) and their corresponding ranges are rather small, allowing for a grid search where each combination is tried out exhaustively.

### Dashboard for physicians

**Fig. 4:**
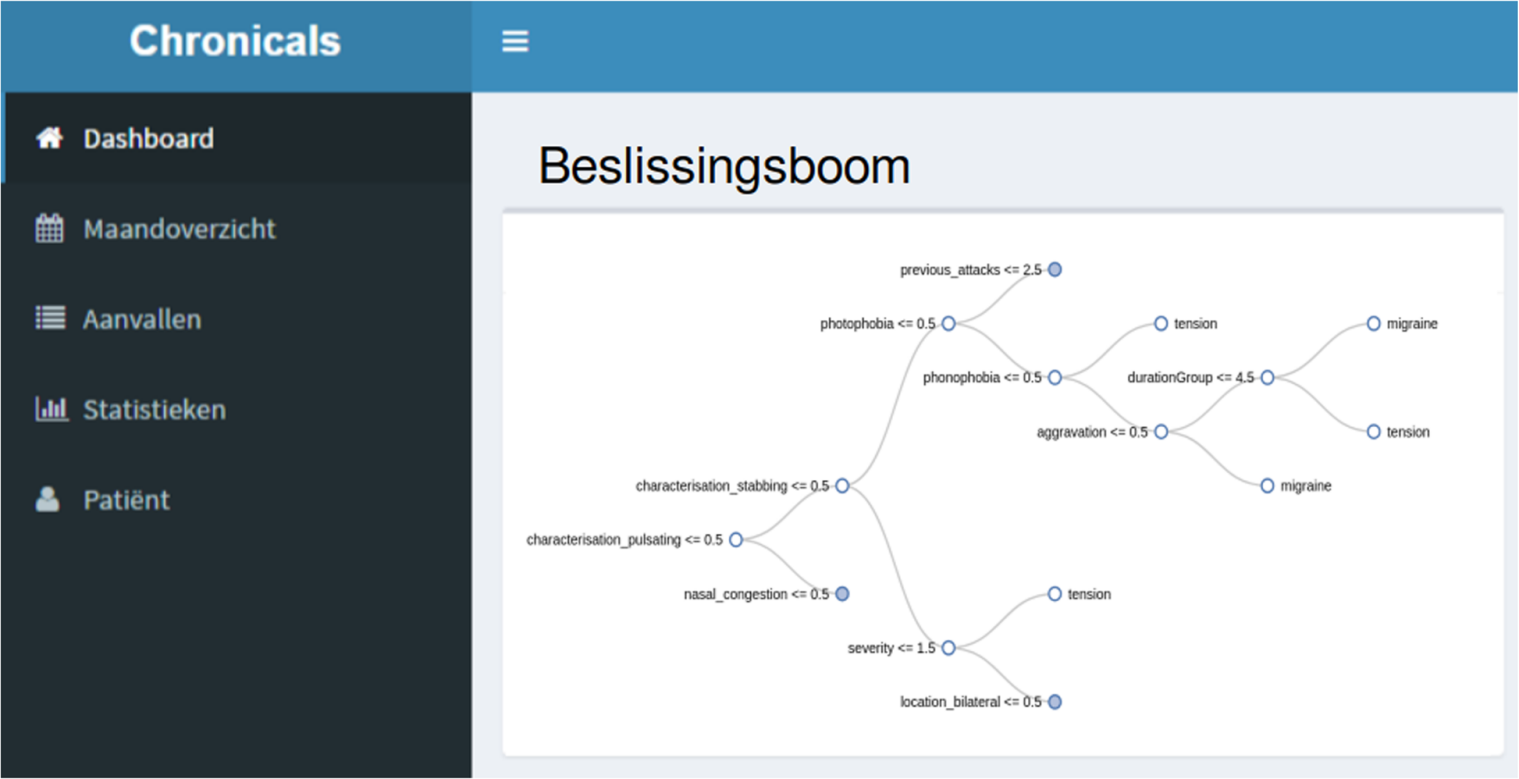
Screenshot of the developed dashboard for physicians: inspecting the decision tree

An important part of the platform is the presentation of both the collected data and the decision trees, induced from this data, towards the physicians. To achieve this, a responsive web application has been developed which physicians can use to prepare a consultation with a patient or as a support while forming a diagnosis. As done for the mobile application, requirements were constructed in consultation with neurologists. The dashboard has been developed within the Java Spring framework [57]. An example of one of pages displaying the inducted decision tree to a physician is shown in Fig. 4.

### Data store and API

All three components share the same back-end. The data from both the mobile and web application are stored in a MongoDB [58] and exposed through a REST API. A MongoDB was preferred over a SQL database because of its flexibility since it does not require the database scheme to be defined prior to storing data in it. This is especially useful in earlier (research) phases, where many variables still tend to get introduced.

### Evaluation setup

In what now follows, we describe the setup for three experiments: (i) comparing different supervised classification techniques on a publicly available dataset, called migbase, (ii) investigating several over-sampling techniques to combat data imbalance, and (iii) an evaluation of different feature extraction algorithms, based on metrics that express the similarity to each class concept. All the code to generate the results was written in Python 3.

#### Data collection and user testing

For a period of four months, every headache patient visiting the neurology department of the University Hospital of Ghent, and diagnosed with a primary headache disorder, was invited to participate in a study wherein both the current paper calendars as well as our mobile application had to be used until the next consultation. Prior to this study, a request (B670201627535, project EC/2016/0172) was submitted to the ethical committee and approved. Before participating, an informed consent was signed and the application was shown and explained to the patient. The goal of this study was two-fold. On the one hand, we could collect initial data for further data analysis. On the other hand, the users could be asked for feedback, such that we could improve our mobile application in terms of usability and functionality.

#### Migbase dataset

Currently, the amount of data collected with our mobile headache journal is not large enough yet to generate statistically significant results. Therefore, an already-existing dataset, called migbase, has been used to generate the results in subsequent sections [59]. This dataset contains answers to questionnaires of 849 different patients from three different hospitals in Turkey. Each sample represents aggregated information from all questionnaires per patient and consists solely of discrete attributes. Each sample is labeled with one of the three categories of primary headache disorders: migraine (71.73*%* of all samples), tension-type headache (21.67*%*) or cluster headache (6.60*%*). A summary of the variables in the migbase dataset can be found in Table 1. Some of the variables, such as the symptoms tinnitus and hypacusia, only had one unique value for all samples, and could thus be discarded.

**Table 1:** The different variables of the migbase dataset, each of the symptoms is a binary variable

Variable	Migbase	Chronicals
Disorder	Migraine, cluster, tension	Migraine with or without aura, cluster, tension
Duration	A: 0-4 seconds	Continuous value (sec. between start and end time)
	B: 5-119 seconds	
	C: 120-239 seconds	
	D: 240-899 seconds	
	E: 900-1799 seconds	
	F: 1800-10799 seconds	
	G: 10800-14399 seconds	
	H: 14400-259199 seconds	
	I: 259200-604799 seconds	
	J: 604800+ seconds	
Location	Unilateral, bilateral, orbital	Frontal (right, mid, left), parietal (right, mid, left), temporal (right, left), occipital (right, mid, left), cervical (right, mid, left), orbital (right, left), mandibular (right, left), maxillar (right, left)
Headache days	< 1; 1−14; 7−365; > 14; > 365, none	Number of days a headache was registered
Severity	Mild, moderate, severe	Scale from 1 to 10
Characterization	Pressing, pulsating, stabbing	Pressing, pulsating, stabbing
Previous attacks	2−4; 5−9; 10−19; 20+	Number of headaches registered
Aura duration	None, hour, day	Derived from duration of headaches with aura symptoms
Symptoms	Nausea, vomiting, photophobia, phonophobia, aggravation (by movement), conjunctival injection, lacrimation, pericranial, nasal congestion, rhinorrhoea, eyelid oedema, forehead and facial sweating, miosis, ptosis, speech disturbance, dysarthria, hemiplegic, visual symptoms, sensory symptoms, homonymous symptoms, agitation, motor weakness, vertigo, tinnitus, hypacusia, diplopia, ataxia, decreased consciousness, nasal symptoms, paraesthesias, aura development, headache with aura	Nausea, vomiting, photophobia, phonophobia, aggravation (by movement), lack of appetite, conjunctival injection, lacrimation, nasal congestion, rhinorrhoea, eyelid oedema, forehead and facial sweating, miosis, ptosis, speech disturbance, visual symptoms, sensory symptoms, motor weakness, facial flushing, aural fulness
Triggers	n/a	Alcohol, sleep deficit, stress, menstruation, fatigue, food, warmth, noise, light

It is important to note that almost all of the variables in the migbase dataset can also be collected with our mobile application. The mapping from the migbase variables to our variables can be found in Table 1 as well.

#### Comparison of classifier techniques

While decision trees possess excellent interpretability, we still need to assess the deficit in terms of predictive performance, specifically in the headache diagnosis domain. Therefore, we compared five decision tree-based algorithms with Neural Network (NN) from the Keras library [60] and Support Vector Machine (SVM), Logistic Regression (LR) and KNN from the scikit-learn library [61]. The decision tree-based techniques are: Classification And Regression Tree (CART) [62] (scikit-learn), C4.5 [63] (Orange [64]), GENetic Extraction of a Single Interpretable Model (GENESIM) [65], Random Forest (RF) [66] (scikit-learn) and eXtreme Gradient Boosting (XGB) [67]. Both CART and C4.5 are naive, top-down induction algorithms. RF and XGB are ensemble techniques, which construct a collection of different decision trees and use these to form a final prediction. These techniques have been shown to outperform their naive counterparts, both theoretically and empirically [68], but at a cost of having much lower interpretability. Finally, the GENESIM technique constructs a large ensemble (constructed using all aforementioned decision tree techniques) and converts this into one decision tree, retaining as much of the positive properties of the ensemble as possible while being fully interpretable. It should also be noted that the CART algorithm was extended with error-based pruning to increase generalization capability [69]. A genetic feature selection algorithm was applied in order to discard uninformative features and to enhance generalization capability of the predictive model. Hyper-parameters were tuned using a grid search for all algorithms, except for XGB, RF and NN as their hyper-parameter space is too large, making a brute-force search computationally infeasible. For XGB and RF, Bayesian optimization was used and the Hyperas [70] library was used to tune the NN topology and other hyper-parameters. Predictions were generated by applying 5-fold cross-validation in a stratified fashion. We report two evaluation metrics. On the one hand, we report the mean accuracy score across the five folds and the corresponding standard deviation. The accuracy score is defined as:


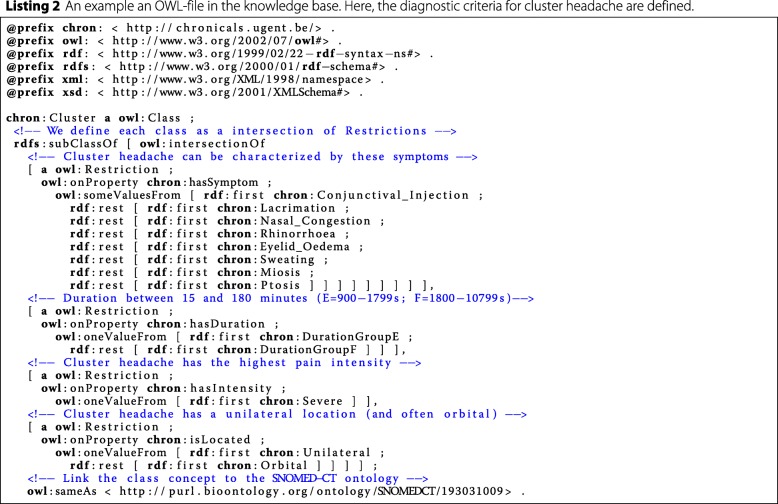

1 $$ \text{accuracy} = \frac{1}{N}*\sum\limits_{1}^{N}\mathbbm{1}_{\widehat{y_{i}} = y_{i}}  $$

with *N* the dataset size, $\mathbbm {1}$ the identity function, $\widehat {y_{i}}$ the prediction for sample *i* and *y*_*i*_ the label of sample *i*. The main advantage of this metric is that it is interpretable, since it intuitively depicts the fraction of correctly classified instances. On the other hand, we also report the mean unweighted Cohen’s kappa score [71] across the five folds and its standard deviation, for its ability to give an objective score, even when the data is imbalanced. The kappa-score (*κ*-score) is defined as:

2 $$ \kappa = \frac{p_{o} - p_{e}}{1-p_{e}}  $$

with *p*_*o*_ the empirical probability of agreement between two annotators on the labels assigned to the samples, which corresponds to the accuracy (1), and *p*_*e*_ the expected agreement when labels are assigned randomly:

3 $$ p_{e} = \frac{1}{N^{2}}\sum\limits_{c=1}^{C}|\{\widehat{y_{i}}\ |\ \widehat{y_{i}} = c\}|*|\{y_{i}\ |\ y_{i} = c\}|  $$

with *C* the number of classes and |{*y*_*i*_|*y*_*i*_=*c*}| or $\left |\left \{\widehat {y_{i}} | \widehat {y_{i}} = c\right \}\right |$ the number of samples or predictions labeled with *c* respectively.

#### Data balancing with prior knowledge

We compared the knowledge-driven oversampling method to SMOTE, ADASYN, a baseline where no sampling techniques are applied and using adjusted weights, where samples from the minority class are given higher weight in the calculation of the split criterion of the induction technique. For the over-sampling techniques, artificial samples were generated such that the number of samples in each class was equal. We then generated predictions using the CART algorithm from scikit-learn on the transformed data with 5-fold cross-validation. No feature selection, pruning or hyper-parameter tuning was applied in order to reduce the required computational time per run. In total, we ran 100 simulations and measured the mean and corresponding standard deviation across these simulations of the following metrics: (i) sensitivity and specificity for each class, (ii) the total accuracy, and (iii) the unweighted Cohen’s *κ*-score. Then, bootstrap testing was applied to test whether the results from two sampling techniques did not stem from the same underlying distribution.

#### Knowledge graph kernels

**Fig. 5:**
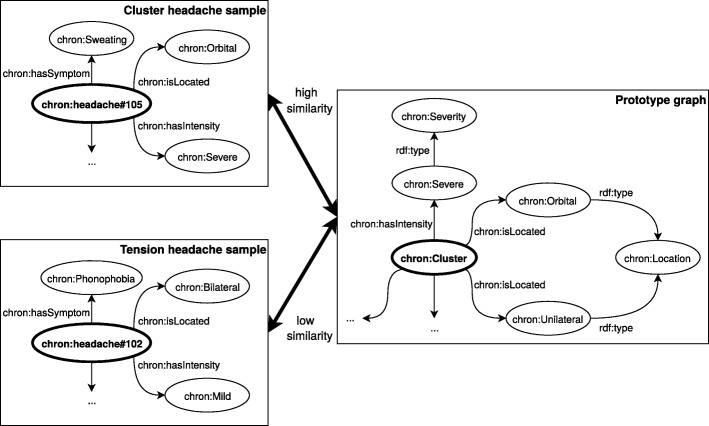
The methodology to calculate similarities between semantically annotated samples and class concepts in our knowledge base

We implemented the fast approximation of WF, proposed by de Vries et al. [50], in Python on top of the rdflib [72] package. Then, we created a knowledge graph for each class concept by taking the union of all values from each restriction, as can be seen in Listing ??, and removing all triples that contain label information (such as triples containing the chron:isType property) in both the annotated samples and our knowledge base. An example, again for the cluster headache class, is depicted in Fig. 5. We compared the WF kernel with a data-driven RBF kernel by measuring the predictive performance when no machine learning classification technique is used, by just simply predicting the class with minimum distance. Moreover, we also compared them to each other by appending the calculated features to our feature vectors and fitting a decision tree from scikit-learn (CART) with no hyper-parameter tuning, pruning or feature selection (again to reduce the required computational time). We applied the same setup as before: 100 simulations with 5-fold cross-validation where we calculated the accuracy and *κ*-score. Again, both the mean and standard deviation, calculated across the different simulations are reported. Afterwards, bootstrap testing was again applied to test whether the values from two techniques did not stem from the same underlying distribution.

## Results

### Comparison of classifier techniques

The accuracies of the different algorithms can be found in Table 2. As we can see, all techniques, including the decision tree induction algorithms, perform very well on the migbase dataset and are competitive to each other, both in terms of global accuracy and the Cohen *κ*-score.

**Table 2:** The different techniques with their corresponding accuracy and *κ*-score on the migbase dataset

Algorithm	Accuracy	Cohen **κ**
GENESIM	0.983510±0.0095	0.958342±0.0237
c4.5	0.981148±0.0087	0.957122±0.0188
RF	0.981148±0.0087	0.957091±0.0189
LR	0.979992±0.0079	0.953758±0.0181
XGB	0.978781±0.0080	0.951446±0.0180
SVM	0.977556±0.0122	0.948858±0.0273
KNN	0.976463±0.0144	0.945615±0.0333
CART	0.976435±0.0065	0.946280±0.0141
NN	0.951250±0.0189	0.916672±0.1471

### Data balancing with prior knowledge

The sensitivity and specificity scores for each class individually and the total accuracy can be found in Table 3. The mean accuracy and kappa scores can be found in Table 4. The margin for improvement is rather small, since the accuracy of the induced decision tree is already 97.85*%* originally. Still, we can observe that using adjusted weights or ADASYN deteriorates both the accuracy and the *κ*-score of the baseline, with statistical significance (*ρ*≤0.05), while using Prior Knowledge improves both metrics with statistical significance. If we look at the sensitivity and specificity of each class individually, we notice that using Prior Knowledge improves either the sensitivity or specificity with statistical significance while having a similar score for the other metric, compared to the baseline, thus improving the predictive performance for each class individually.

**Table 3:** The mean sensitivity and specificity scores with corresponding standard deviations for each class individually on the migbase dataset for the original training set and the transformed datasets obtained using three over-sampling techniques

Technique	Migraine		Tension		Cluster	
	Sensitivity	Specificity	Sensitivity	Specificity	Sensitivity	Specificity
Prior knowledge	**0.9848±0.00**3^+^	0.9753±0.006	0.9682±0.007	**0** **.** **9** **8** **7** **5** **±** **0** **.** **0** **0** **3** ^+^	**0.9775±0.01**1^+^	0.9973±0.002
ADASYN	0.9839±0.003	**0.9771±0.00**5^+^	0.9683±0.007^−^	0.9836±0.003^−^	0.9421±0.022^−^	0.9969±0.002
SMOTE	0.9845±0.003^+^	0.9767±0.006^+^	**0.9721±0.00**8^+^	0.9845±0.003	0.9307±0.024^−^	0.9967±0.002
Sample weight	0.9830±0.003	0.9742±0.007	0.9696±0.008	0.9827±0.003^−^	0.9250±0.024^−^	0.9969±0.002
None	0.9834±0.003	0.9744±0.006	0.9695±0.008	0.9850±0.003	0.9556±0.021	**0** **.** **9** **9** **7** **4** **±** **0** **.** **0** **0** **2**

A cell is marked as.^+^ or.^−^ if the result is a statistically significant (*ρ*≤0.05) improvement or detriment respectively compared to the baseline (None), according to a bootstrap test

**Table 4:** The mean accuracies and *κ*-scores on the migbase dataset for the original training set and the transformed datasets obtained using three over-sampling techniques

Technique	Accuracy	Cohen **κ**
Prior knowledge	**0.9807±0.002**5^+^	**0.9558±0.005**7^+^
ADASYN	0.9775±0.0026^−^	0.9490±0.0058^−^
SMOTE	0.9782±0.0034	0.9501±0.0077
Sample weight	0.9762±0.003^−^	0.9457±0.0069^−^
None	0.9785±0.0029	0.9508±0.0066

A cell is marked as.^+^ or.^−^ if the result is a statistically significant (*ρ*≤0.05) improvement or detriment respectively compared to the baseline (None), according to a bootstrap test

### Knowledge graph kernels

The predictive performance metrics for the different feature extraction techniques are listed in Table 5. We notice that applying solely the RBF kernel achieves a better predictive performance than the original feature set, but with no statistical significance. On the other hand, if we append features extracted using this kernel to our original dataset, we confuse our classifier and get a detriment with statistical significance. This is not the case when we append the calculated features using the WF kernel, where we notice a slight improvement, although with no statistical significance (*ρ*>0.05). Moreover, we are able to achieve an accuracy rate of 93.39% with the WF kernel, without using a single labeled sample or a machine learning algorithm.

**Table 5:** The accuracy rates on the public migbase dataset for the different feature extraction techniques

Technique	Accuracy	Cohen **κ**
Only RBF	0.9788±0.0115	0.9522±0.0251
Original + RBF	0.9692±0.0167^−^	0.9303±0.0368^−^
Only WF	0.9339±0.0384^−^	0.8588±0.0809^−^
Original + WF	0.9795±0.0155	0.9534±0.0342
Original + WF + RBF	0.9692±0.0150^−^	0.9301±0.0323^−^
Original	0.9784±0.0107	0.9508±0.0237

A cell is marked as.^+^ or.^−^ if the result is a statistically significant improvement or detriment respectively compared to the baseline (Original), according to a bootstrap test

## Discussion

In total, 32 patients, that were diagnosed with a primary headache disorder by a physician, used the application and 456 headaches were registered in the system. We received positive feedback from both users and physicians, but no formal usability study, has been conducted. While a mobile application has its advantages over a paper calendar, including being available at any time and place, and not having to worry about losing it, such a study is required to further clarify the usability of the application as compared to the paper diary.

The fact that decision trees are competitive to, and even outperform some of the other techniques can be explained by the analogy between decision trees and the ICHD document, which can both be boiled down to if-then rules. Because of their excellent interpretability, they therefore form a perfect match as a decision support tool. While GENESIM achieves the best accuracy and Cohen’s *κ*-score, the difference is rather small and the time needed to train the model is several orders of magnitude higher than the other techniques (hours as opposed to minutes). Therefore, C4.5 or CART are more suited candidates in practice. One possible improvement would thus be to reduce the computational complexity of the GENESIM technique.

Using prior knowledge to balance the class distributions in the dataset enhances both the predictive performance for each minority class as the global predictive performance as opposed to data-driven techniques. Moreover, transparency is enhanced since the knowledge base in our system, which is constructed using knowledge defined by experts, impacts the resulting predictive model. This makes it an ideal pre-processing step for medical or other critical domains. Finally, since the classification which uses only similarity scores calculated by the WF-kernel, in an unsupervised fashion, performs not much worse than when a decision tree is fit on all data, this technique seems ideally suited to solve a ‘cold start’ problem (when too few labeled examples are available).

While the migbase dataset provides an opportunity for us to test the feasibility of the automated diagnosis support module, the data quality is very high and the dataset only contains three classes. It is therefore of primal importance, before deploying the proposed system in a real clinical setting, to re-evaluate the system on a larger, more realistic dataset. This dataset should contain a greater number of different classes (preferably up to the third digit of the ICHD classification). Moreover, detection of red flag signs that could indicate a secondary headache should be built in the system as well [73].

The semantic encoding and decoding phase in the proposed automated diagnosis module could be made redundant if we extend machine learning algorithms to deal directly with semantically annotated data, a research domain still in its infancy [74, 75].

We could facilitate trigger management by extending our mobile application in order to automatically detect possible triggers and motivate users to adjust their lifestyle to avoid these possible triggers.

## Conclusion

In this paper, we presented a proof-of-concept of an end-to-end decision support system in order to diagnose and follow-up primary headache patients. We believe that the deployment of such a system in a neurology department could significantly increase the efficiency of the different processes, thus possibly reducing health-care costs. The decision support system consists of three large components and a shared back-end: a mobile application for the patients, a web application to visualize the collected data to the physicians and an automated diagnosis module. For the automated diagnosis module, decision trees are an ideal candidate as the modeling technique since they possess excellent comprehensibility and because their predictive performances are shown to be competitive to and even outperform other techniques. Moreover, we show the potential of applying both data-driven as knowledge-driven techniques in each step of the machine learning pipeline by presenting: a technique to balance the dataset which outperforms the current state-of-the-art on the migbase dataset and an unsupervised feature extraction technique, based on WF kernels that measure graph similarity. Furthermore, on top of the gain in predictive performance, transparency and interpretability are enhanced since knowledge, provided by experts, is directly incorporated in the machine learning algorithm, which can lower the threshold of adaption by physicians. Future work includes re-running all experiments on the data collected by our application and with a more fine-grained classification, once more data is available. Furthermore, we would like to extend our mobile application with user behavioral pattern recognition in order to automate event logging as well as trigger detection for headache disorders. This way, automated feedback can be given to patients in order to adjust their lifestyle to try avoiding these triggers and hence reduce the amount of headache attacks. Moreover, current existing machine learning techniques could be extended such that they are able to directly deal with semantically annotated data, alleviating the need for the semantic encoding and decoding phase in the automated diagnosis support module.

## Abbreviations

adasyn: ADAptive SYNthetic sampling approach
cart: Classification And Regression Tree
genesim: GENetic Extraction of a Single, Interpretable Model
guide: Generalized Unbiased Interaction Detection and Estimation
ichd: International Classification of Headache Disorders
ism: Interpretable Single Model extraction
knn: K-Nearest Neighbor
lr: Logistic Regression
nb: Naive Bayes
nn: Neural Network
owl: Web Ontology Language
quest: Quick Unbiased Efficient Statistical Tree
rbf: Radial Basis Function
rdf: Resource Description Framework
rf: Random Forest
smote: Synthetic Minority Oversampling TEchnique
svm: Support Vector Machine
wf: Weisfeiler-Lehman
who: World Health Organization
xgb: eXtreme Gradient Boosting
